# Identification of biomarkers in macrophages of atherosclerosis by microarray analysis

**DOI:** 10.1186/s12944-019-1056-x

**Published:** 2019-05-01

**Authors:** He-ming Huang, Xin Jiang, Meng-lei Hao, Meng-jie Shan, Yong Qiu, Gai-feng Hu, Quan Wang, Zi-qing Yu, Ling-bing Meng, Yun-yun Zou

**Affiliations:** 1 Geriatric Department, Shenzhen People’s Hospital, The Second Clinical Medical College of Jinan University, The First Affiliated Hospital of Southern University of Science and Technology, Shenzhen, 518020 People’s Republic of China; 2 Department of Geriatric Medicine, Qinghai University, Xining, Qinghai 810016 People’s Republic of China; 3 MOH Key Laboratory of Systems Biology of Pathogens, Institute of Pathogen Biology, Chinese Academy of Medical Sciences and Peking Union Medical College, Beijing, 100730 People’s Republic of China; 4 Anesthesiology Department, Beijing Hospital, National Center of Gerontology, No.1 Dahua Road, Dong Dan, Beijing, 100730 People’s Republic of China; 5 Department of Cardiology, Beijing Hospital, National Center of Gerontology, No.1 Dahua Road, Dong Dan, Beijing, 100730 People’s Republic of China; 6 Pneumology Department, Beijing Hospital, National Center of Gerontology, No.1 Dahua Road, Dong Dan, Beijing, 100730 People’s Republic of China; 7 Neurology Department, Beijing Hospital, National Center of Gerontology, No.1 Dahua Road, Dong Dan, Beijing, 100730 People’s Republic of China; 8 The Fifth Ward of Ophthalmology Department, Shenzhen Eye Hospital, Shenzhen, 518040 People’s Republic of China

**Keywords:** Atherosclerotic cardiovascular disease, Atherosclerosis, Macrophages, Hub genes, Bioinformatics

## Abstract

**Background:**

Atherosclerotic cardiovascular disease (ASCVD) refers to a series of diseases caused by atherosclerosis (AS). It is one of the most important causes of death worldwide. According to the inflammatory response theory, macrophages play a critical role in AS. However, the potential targets associated with macrophages in the development of AS are still obscure. This study aimed to use bioinformatics tools for screening and identifying molecular targets in AS macrophages.

**Methods:**

Two expression profiling datasets (GSE7074 and GSE9874) were obtained from the Gene Expression Omnibus dataset, and differentially expressed genes (DEGs) between non-AS macrophages and AS macrophages were identified. Functional annotation of the DEGs was performed by analyzing the Gene Ontology and Kyoto Encyclopedia of Genes and Genomes databases. STRING and Cytoscape were employed for constructing a protein–protein interaction network and analyzing hub genes.

**Results:**

A total of 98 DEGs were distinguished between non-AS macrophages and AS macrophages. The functional variations in DEGs were mainly enriched in response to hypoxia, respiratory gaseous exchange, protein binding, and intracellular, ciliary tip, early endosome membrane, and Lys63-specific deubiquitinase activities. Three genes were identified as hub genes, including *KDELR3*, *CD55*, and *DYNC2H1*.

**Conclusion:**

Hub genes and DEGs identified by using microarray techniques can be used as diagnostic and therapeutic biomarkers for AS.

## Background

Atherosclerotic cardiovascular disease (ASCVD) refers to a series of diseases caused by atherosclerosis (AS), including acute coronary syndromes, myocardial infarction, stable and unstable angina, revascularization of coronary or other arterial blood vessels, stroke, transient ischemic attack, and arterial AS sclerosing peripheral arterial disease [1]. ASCVD is one of the most important causes of death in elderly patients at home and abroad [2]. According to the World Health Organization, 17 million people died of ASCVD worldwide in 2008, accounting for 30% of the total disease mortality, 80% of which occurred in low- and middle-income countries. It is predicted that, by 2030, the number of deaths due to ASCVD will increase to 23.3 million. The burden of ASCVD is increasing, and the disease has become a major public health problem. It is, therefore, particularly important to strengthen current research mechanisms for this disease [3].

AS is an arterial disease associated with dyslipidemia and changes in the components of blood vessel walls. It mainly affects large and medium-sized arteries—especially those in the heart, brain, kidneys, and other organs—and can cause ischemic changes [4]. The currently recognized risk factors for AS include smoking, alcohol, genetic factors, hypertension, and hyperlipidemia. Some diseases that cause secondary hyperlipidemia (e.g., diabetes and nephrotic syndrome) and other factors (e.g., age and obesity) [5]. The pathogenesis of AS has been a hot topic in the medical world. For a long time in the past, AS was considered as passive fat deposition in blood vessel walls. We now know that AS is a chronic inflammatory disease caused by lipids, especially low-density lipoproteins (LDLs), and white blood cells. Increased plaque inflammatory response can induce local protein hydrolysis, resulting in plaque rupture and thrombosis, ultimately leading to the development of ASCVD [6]. This phenomenon formed the basis for the “inflammatory response theory”.

Regarding the theory of inflammatory response, the statement by Ross [7] that “AS is an inflammatory disease” is recognized by most scholars. The development of AS lesions is mainly influenced by network relationships between endothelial cells, smooth muscle cells, macrophages, and T lymphocytes. Macrophage production leads to the secretion of several growth-stimulating factors, which cause changes in the phenotype of blood vessels. The phenotype of smooth muscle cells changes from their original normal contractile type to the naive synthesis type; these cells then proliferate, migrate to the endometrium, synthesize and secrete stimulating factors, and finally stimulate macrophages, leading to continuous macrophage proliferation. T lymphocytes continue to proliferate and replicate like macrophages. Macrophages and T lymphocytes play an important role in AS. Therefore, reducing cholesterol accumulation in macrophages and inhibiting vascular wall inflammation are important for the prevention and treatment of AS [8].

An increasing number of studies have shown that the role of macrophages in the development of AS is closely related to mutation and abnormal expression of genes [9–11]. Higashi et al. indicated that the macrophage insulin-like growth factor-1 receptor signaling pathway inhibits the aggregation of macrophages and foam cells during the development of atherosclerotic lesions and reduces plaque instability, providing a novel mechanism for antiatherogenic research [9]. In addition, Wang et al. demonstrated the critical role of C1q/TNF-related proteins-1 (CTRP1) in linking the dysregulation of lipid metabolism and inflammatory responses in macrophages. Stimulation of CTRP1 significantly enhances the secretion of proatherogenic factors, including monocyte chemoattractant protein-1, tumor necrosis factor-α (TNF-α), interleukin-1 beta, and interleukin-6 (IL-6) [10]. Moreover, another study has suggested that deletion or low expression of interferon regulatory factor 2-binding protein 2 (IRF2BP2) in macrophages can aggravate AS [11]. However, because of the lack of strategies for timely detection, dynamic monitoring, and effective control of macrophages, it has not been possible to effectively alleviate atherosclerotic cardiovascular and cerebrovascular diseases worldwide. Therefore, it is particularly important to identify the precise genetic targets associated with macrophages in the development of AS [12].

With the rapid development of science and technology in the past few years, bioinformatics and microarray technologies have become widely employed for screening and predicting disease gene targets [13]. These technologies can help researchers identify differentially expressed genes (DEGs) and potentially different pathways between non-AS and AS macrophages.

This study aimed to employ bioinformatics tools for screening and identifying molecular targets in AS macrophages. Two human gene expression profiling datasets were downloaded from the Gene Expression Omnibus (GEO) dataset and analyzed in order to identify DEGs between non-AS and AS macrophages. Then, the molecular mechanisms underlying the development of AS macrophages were researched by enrichment and protein–protein interaction (PPI) network analyses. In the end, 98 DEGs and 3 hub genes were conclusively authenticated.

## Methods

### Access to public-domain data

GEO (http://www.ncbi.nlm.nih.gov/geo) is an open functional genomics database of high-throughput resources, including data from microarray, gene expression, and gene chip analyses. For this study, we downloaded two expression profiling datasets (GSE7074 and GSE9874) from GEO. The probes were transformed into homologous gene symbols by using the platform’s annotation data. All macrophage data were derived from in silico studies. The GSE7074 dataset contains 8 non-AS-macrophage samples from non-atherosclerotic tissues and 8 AS-macrophage samples from atherosclerotic plaques of the carotid artery. The GSE9874 dataset contains 15 non-AS-macrophage samples from subjects without AS and 15 AS-macrophage samples from atherosclerotic tissues.

### DEGs identified by GEO2R

GEO2R (http://www.ncbi.nlm.nih.gov/geo/geo2r), an online data analysis tool, was used for screening DEGs between non-AS- and AS-macrophage samples. After setting up differential experimental groups for one of the GEO series, GEO2R was instructed to execute a command for comparing differential classifications in order to identify DEGs. When the gene symbol corresponded to the probes, the data were usually considered valuable and were reserved. The level of statistical significance was set at *P* ≤ 0.05.

### Functional annotation of DEGs by GO and KEGG analysis

DAVID (https://david.ncifcrf.gov/home.jsp; version 6.8), an online analysis tool suite for integrated discovery and annotation, mainly provides typical batch annotation and gene–GO term enrichment analysis for highlighting the most relevant GO terms associated with a given gene list. Kyoto Encyclopedia of Genes and Genomes (KEGG) (https://www.kegg.jp/), one of the most commonly used biological information databases in the world, aims to understand advanced functions in biological systems. At a molecular level, KEGG specifically integrates a large number of practical program database resources from high-throughput experimental technologies. Gene Ontology (GO) is a widely used ontology in bioinformatics; it covers three aspects of biology: cellular component (CC), molecular function (MF), and biological process (BP). In this study, the DAVID online tool was used to perform GO and KEGG analysis of the DEGs. The level of statistical significance was set at *P* < 0.05.

### Construction and analysis of a PPI network

The common DEGs were imported to the online “Search Tool for the Retrieval of Interacting Genes” (STRING; http://string-db.org; version 10.5), which predicted and traced the PPI network. Analysis of interactions between various proteins might provide some novel ideas on the pathophysiological mechanisms of AS development. In this study, a PPI network of DEGs was constructed by using the STRING database with a minimum required interaction score of > 0.4 (medium confidence).

### Mining and analysis of hub genes

Molecular Complex Detection (MCODE; version 1.5.1), a plugin of Cytoscape, can discover tightly coupled regions on the basis of topological principles. In this study, MCODE was used to identify the most important module of the PPI network map. The criteria for MCODE analysis were as follows: degree cutoff, 2; MCODE scores, > 5; max depth, 100; k-score, 2; and node score cutoff, 0.2. Hub genes were identified when the degree cutoff was set at ≥10. Then, the hub genes were submitted for GO and clustering analysis by using OmicShare (http://www.omicshare.com/tools), an open data analysis platform.

## Results

### DEGs identified between non-AS- and AS-macrophages samples

**Fig. 1:**
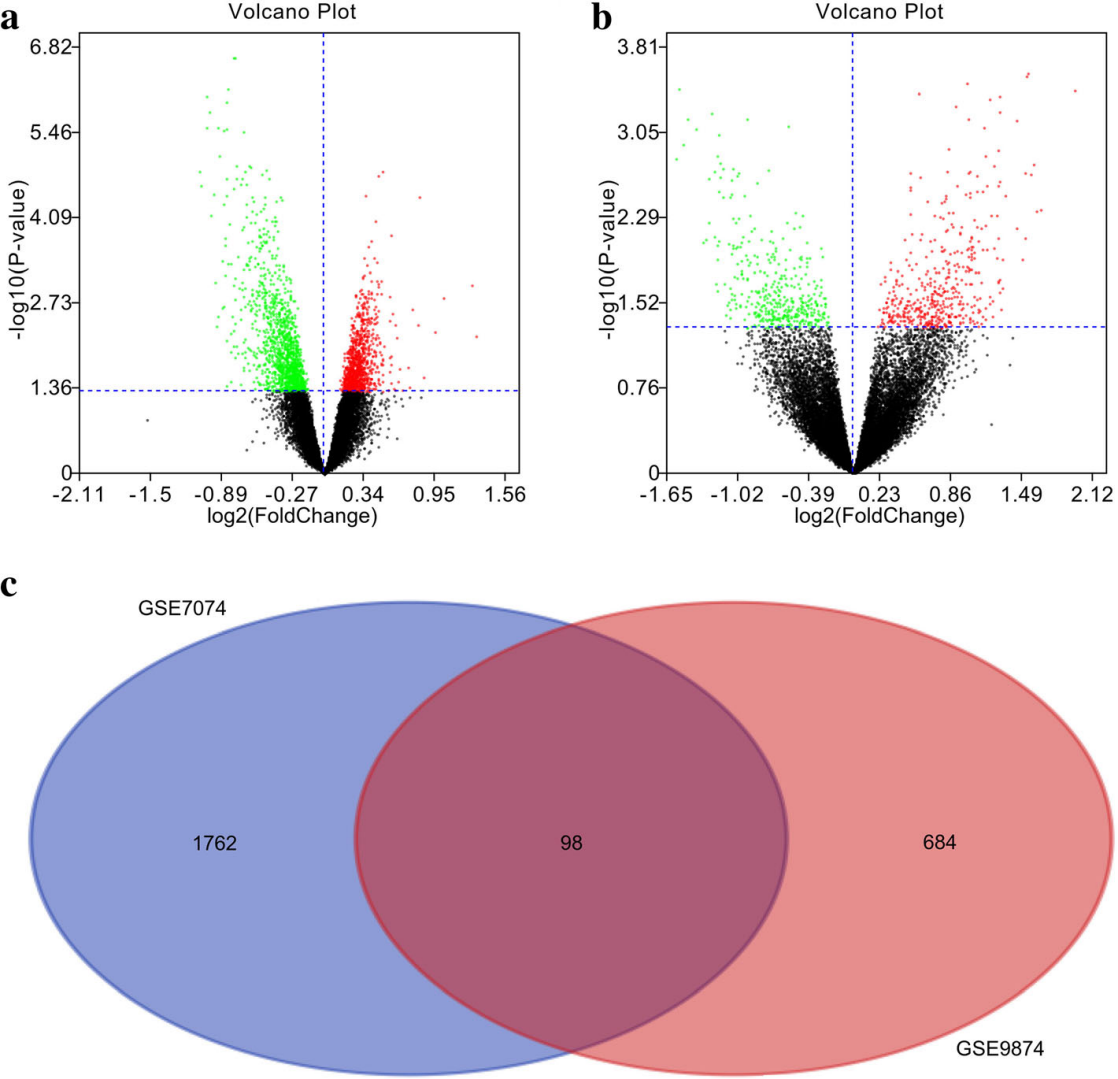
Identification of DEGs between non-AS and AS macrophages. Volcano plots presenting the differences between non-AS and AS macrophages after analysis of the (**a**) GSE7074 and (**b**) GSE9874 datasets by using GEO2R; (**c**) Venn diagram showing the 98 DEGs identified simultaneously in the GSE7074 and GSE9874 datasets. DEG: differentially expressed genes; AS, atherosclerosis

After analyzing the GSE7074 and GSE9874 datasets by using GEO2R, the differences between non-AS- and AS-macrophage samples were plotted in the form of volcano plots (Figs. 1 a,b). Then, these results were standardized, and the DEGs between non-AS and AS macrophages (1860 in GSE7074 and 782 in GSE9874) were identified. A Venn diagram showed that the two datasets contained 98 DEGs in common (Fig. 1 c).

### Functional annotation of DEGs by GO and KEGG analysis

The results of GO analysis demonstrated that the variations in BPs, CCs, and MF of the DEGs were mainly enriched in negative regulation of TOR (target of rapamycin) signaling, response to hypoxia, respiratory gaseous exchange, thiol-dependentubiquitin-specific protease activity, protein binding, Lys63-specific deubiquitinase activity, and intracellular, ciliary tip, and early endosome membrane activities (Table 1). The results of analysis of KEGG pathways revealed that all DEGs were primarily enriched in the Ras, insulin, ErbB, and gonadotropin-releasing hormone (GnRH) signaling pathways (Table 1).

**Table 1:** GO and KEGG pathway enrichment analysis of DEGs between non-AS macrophages and AS macrophages

Term	Description	Count in gene set	*P*-value
GO:0032007	negative regulation of TOR signaling	3	0.010
GO:0001666	response to hypoxia	5	0.014
GO:0007585	respiratory gaseous exchange	3	0.015
GO:0005622	intracellular	16	0.003
GO:0097542	ciliary tip	3	0.022
GO:0031901	early endosome membrane	4	0.022
GO:0004843	thiol-dependent ubiquitin-specific protease activity	4	0.009
GO:0005515	protein binding	58	0.015
GO:0061578	Lys63-specific deubiquitinase activity	2	0.026
hsa04014	Ras signaling pathway	7	0.003
hsa04910	Insulin signaling pathway	5	0.012
hsa04012	ErbB signaling pathway	4	0.019
hsa04912	GnRH signaling pathway	4	0.021

*GO* Gene Ontology, *KEGG* Kyoto Encyclopedia of Genes and Genomes, *DEGs* differentially expressed genes, *AS* atherosclerosis

### PPI network construction and module analysis

**Fig. 2:**
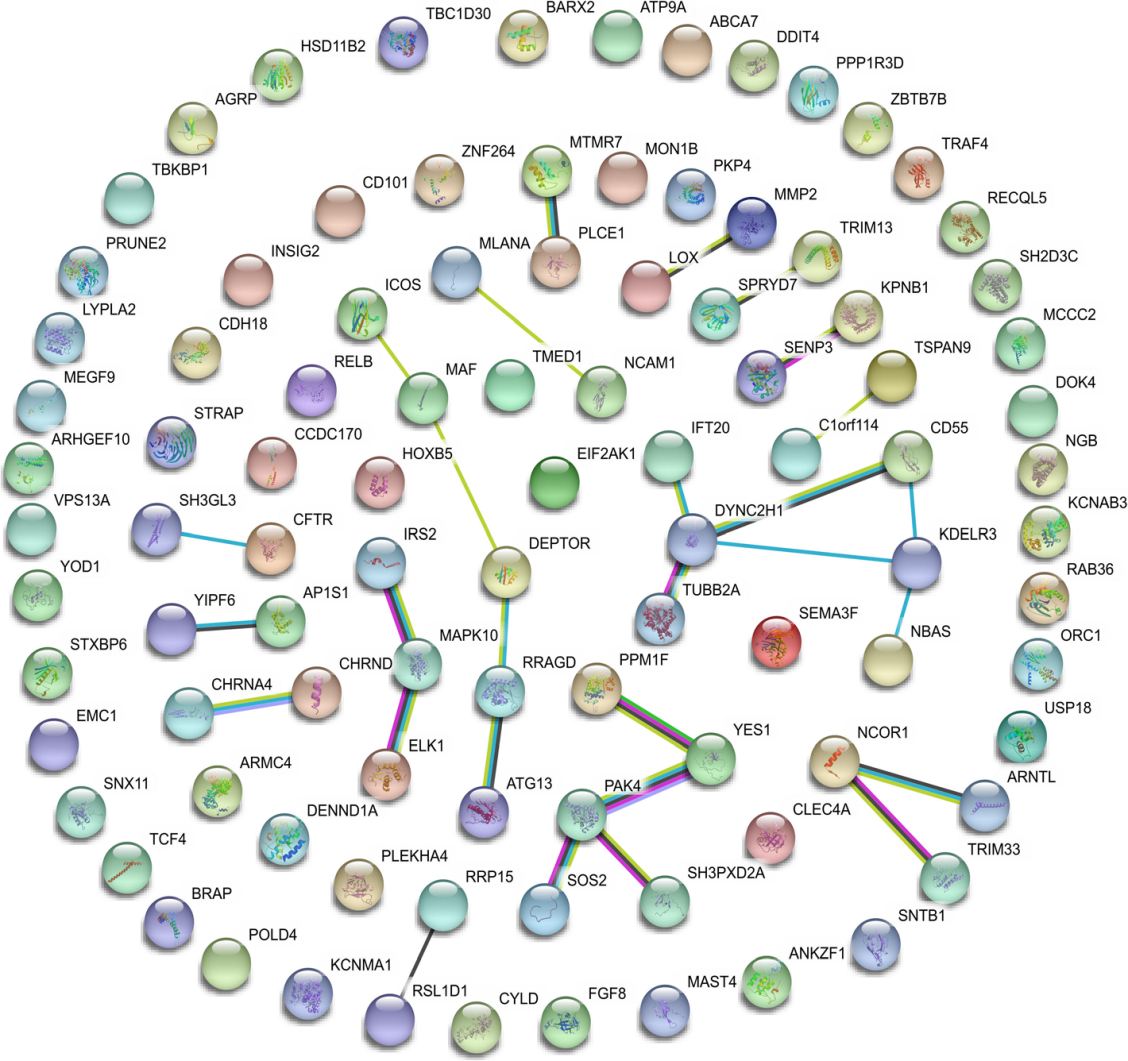
Protein–protein interaction network of differentially expressed genes

**Fig. 3:**
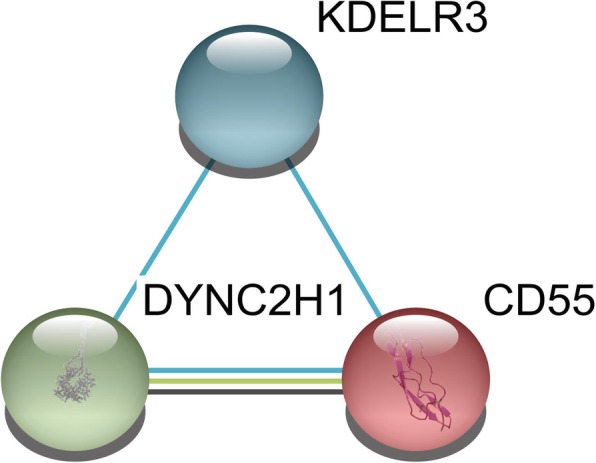
The most significant module identified in the protein–protein interaction network

**Fig. 4:**
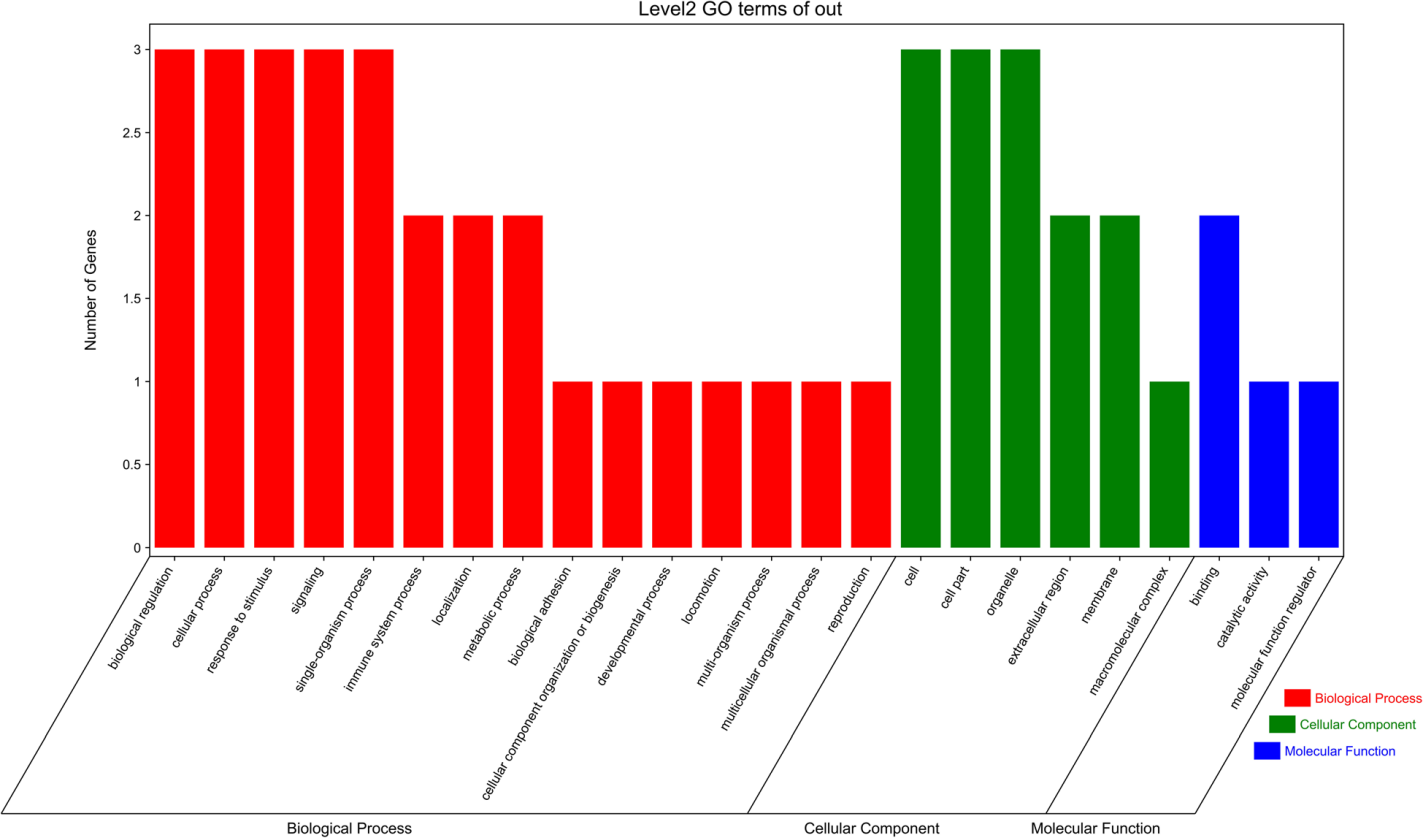
Gene Ontology enrichment analysis of hub genes

A PPI network of the DEGs was constructed (Fig. 2), and the most significant module of the PPI network was identified by using Cytoscape (Fig. 3). Functional analysis of the genes involved in this module was performed by using DAVID. The results showed that the genes in this module were mainly involved in biological regulation, cellular processes, response to stimulus, signaling, cells, cell parts, organelles, extracellular regions, membranes, macromolecular complexes, binding, catalytic activity, and molecular function regulation (Fig. 4).

### Hub gene selection and analysis

**Fig. 5:**
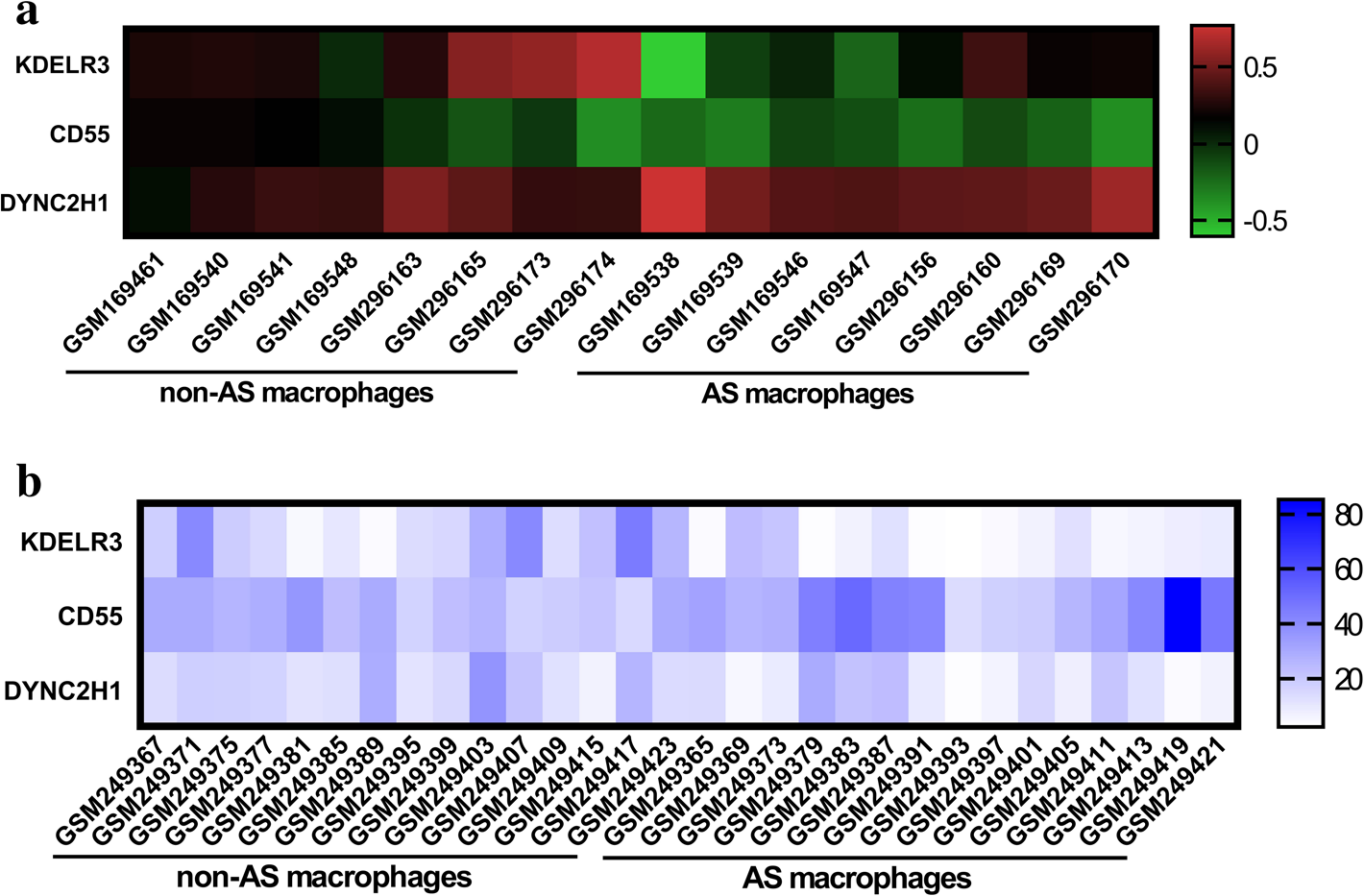
Results of hierarchical clustering showing that the hub genes can differentiate AS and non-AS macrophages in the (**a**) GSE7074 and (**b**) GSE9874 datasets

A total of three genes—*KDELR3* (KDEL endoplasmic reticulum protein retention receptor 3), *CD55* (complement decay-accelerating factor), and *DYNC2H1* (dynein cytoplasmic 2 heavy chain 1)—were identified `as hub genes from the most significant module, with a degree cutoff of ≥10. The names, abbreviations, and functions of these hub genes are shown in Table 2. The results of hierarchical clustering showed that these hub genes could differentiate AS-macrophage samples from non-AS-macrophages samples (Figs. 5 a,b). These hub genes showed the highest node score in the PPI network, suggesting that they might play important roles in the occurrence or progression of AS.

**Table 2:** Summaries for the function of 3 hub genes

No.	Gene symbol	Full name	Function
1	KDELR3	KDEL endoplasmic reticulum protein retention receptor 3	Required for the retention of luminal endoplasmic reticulum proteins. Determines the specificity of the luminal ER protein retention system. Also required for normal vesicular traffic through the Golgi. This receptor recognizes K-D-E-L (By similarity).
2	CD55	CD55 molecule (Cromer blood group)	This gene encodes a glycoprotein involved in the regulation of the complement cascade. Binding of the encoded protein to complement proteins accelerates their decay, thereby disrupting the cascade and preventing damage to host cells. Antigens present on this protein constitute the Cromer blood group system (CROM). Alternative splicing results in multiple transcript variants. The predominant transcript variant encodes a membrane-bound protein, but alternatively spliced transcripts may produce soluble proteins
3	DYNC2H1	Dynein cytoplasmic 2 heavy chain 1	May play a role in transport between endoplasmic reticulum and Golgi or organization of the Golgi in cells (By similarity).

## Discussion

The morbidity and mortality of AS have increased over the years, seriously endangering human health [14]. The pathogenesis of AS is complex and explained by many theories, including “lipid infiltration” and “inflammatory response” [15]. On the one hand, very-low-density lipoproteins, LDLs, and lipoproteins invade and accumulate in the vascular wall, resulting in the thickening of the vascular intima and narrowing of the lumen. Monocytes differentiate into macrophages and phagocytose a large amount of lipids to transform into foam cells, promoting the development of AS [16]. On the other hand, foam cells secrete several inflammatory factors—such as TNF-α, interleukin-1 (IL-1), and IL-6—which further leads to cellular adhesion, infiltration of inflammatory cells, and matrix degradation, resulting in atherosclerotic plaque rupture [15]. Therefore, inhibiting the accumulation of lipids in macrophages and decreasing inflammatory response are critical measures for preventing AS development. Metabolic processes in macrophages are affected by multiple target genes, and a single target gene probably also regulates multiple micro-RNAs, which makes research on the occurrence and development of AS macrophages complex and challenging [17, 18]. It is, therefore, critical to explore the precise molecular mechanism of macrophages in AS and identify reliable therapeutic targets.

With recent developments in biogenetics exploration technologies, studies have demonstrated differential gene expression in several other diseases, which provides ideas for accurate treatment of such diseases [19–21]. For example, four molecular markers associated with the development of gliomas have been identified through biogenetics approaches [19]. A study on hepatocellular carcinoma employed microarray technology to search and analyze a biological database and finally identified 16 hub genes closely related to the development of hepatocellular carcinoma [20]. Another study analyzed the expression levels of 322 immune genes and found that the immune characteristics of patients with neuroblastoma were enhanced; this study finally selected eight immune genes as molecular markers of neuroblastoma [21]. The emergence of biogenetics techniques and their application in prediction of disease target genes provide us new directions and technical support for selecting DEGs related to AS macrophages.

In this study, we used bioinformatics tools to identify DEGs and target molecular markers in AS macrophages. Analysis of the GSE7074 and GSE9874 datasets by using GEO2R helped us identify DEGs between non-AS- and AS-macrophage samples. Both datasets included 98 DEGs in common. Through enrichment analysis, we found that these DEGs were mainly enriched in negative regulation of TOR signaling; response to hypoxia; respiratory gaseous exchange; intracellular, ciliary tip, and early endosome membrane activities; thiol-dependent ubiquitin-specific protease activity; protein binding; Lys63-specific deubiquitinase activity; and the Ras, insulin, ErbB, and GnRH signaling pathways. Furthermore, in the most significant module of the PPI network, three hub genes—*KDELR3*, *CD55*, and *DYNC2H1*—showed the highest score, suggesting that they might have significant roles in the occurrence or development of AS.

*KDELR3*, the third confirmed member of the *KDEL* family, encodes proteins associated with the endoplasmic reticulum (ER) [22]. According to our analysis, *KDELR3* expression in AS macrophages was significantly different from that in non-AS tissues. *KDELR3* can regulate the specific functions of proteins in the ER lumen. However, the ER is the center for the synthesis of important biomacromolecules such as proteins, lipids (e.g., triglycerides), sugars, and nucleic acids. The ER exists in all types of eukaryotic cells except mature mammalian erythrocytes [23]. It is an interconnected lamellar lacunar or tubular system composed of biofilms. The lacunar space between the membranes is called a pool, and it is usually not directly connected with the extracellular lacunae or cytoplasmic matrix. This intracellular membranous piping system constitutes the intracellular transport pathway for materials and also provides a broad reaction area for a variety of enzymes within the cell [23]. The ER is connected with the nuclear and cell membranes and can be divided into smooth and rough ER. Smooth ER has a smooth surface and does not bind ribosomes. It is mainly involved in the synthesis and transportation of steroids and lipids as well as in glucose metabolism and hormone inactivation. Studies have shown that stress is one of the risk factors for AS development [24]. Macrophages activated after stress contain abundant smooth ER, which is involved in lipoprotein processing and modification and lipid synthesis and transport and thus participates in the formation and development of AS. Therefore, we speculate that *KDELR3* expression plays an important role in the development of AS macrophages. Therefore, *KDELR3* could serve as a diagnostic marker of AS.

*CD55* encodes glycoproteins involved in the regulation of complement cascades, which bind to complement proteins and accelerate their own decay, a process that disrupts complement cascades and avoids killing host cells [25]. According to our results, *CD55* expression in AS-macrophage samples was significantly lower than that in non-AS tissues. Complement proteins are a type of serum proteins found in the serum and tissue fluids of humans and vertebrates. They possess enzymatic activity and can mediate immune responses and inflammation after activation [26]. The complement system is activated through three relatively independent but interrelated pathways in order to exert a variety of biological effects, including regulation of phagocytosis, cell lysis, mediation of inflammation, immune regulation, and immune-complex clearance. The complement system can not only enhance phagocytosis and macrophage chemotaxis but also increase vascular permeability. Complement cascade is a process that amplifies weak biological signals and enhances their biological effects in a step-by-step manner [27]. We, therefore, speculate that *CD55* is expressed at low concentrations in AS samples. When the body is stimulated by various endogenous and exogenous stimuli, the *CD55*-encoded glycoprotein fails to block the complement cascade, which results in the enhancement of macrophage chemotaxis and inflammatory response, further aggravating AS. We, therefore, speculate that *CD55* expression plays an important role in the development of AS.

*DYNC2H1* encodes a cytoplasmic dynamic protein which acts as a motor for the axoplasmic transport of cellular cilia and might play an important role in transport between the ER and Golgi apparatus [28]. According to our results, *DYNC2H1* expression levels in AS-macrophage samples were significantly higher than those in non-AS tissues. As mentioned above, when the body is stimulated by stress, macrophages are activated, and a large amount of smooth ER accumulates in the cytoplasm, where it participates in the synthesis and transportation of steroids and lipids [29]. *DYNC2H1* is expressed at high concentrations in AS samples; this *DYNC2H1* upregulation enhances the transportation power of the ER and promotes lipid phagocytosis by macrophages, further advancing the progression of AS. Therefore, we speculate that *DYNC2H1* expression plays an important role in the development of AS. Furthermore, *DYNC2H1* may be used as a therapeutic target for AS in a clinical setting.

The present study has some limitations. The screening of the three key genes related to the development and progression of AS was based on bioinformatics technologies and thus requires further validation in in vitro and in vivo experiments. Therefore, relevant samples should be collected in clinical work, and an animal model of AS should be established to verify the mechanism of action of the three genes in AS macrophages.

## Conclusions

In conclusion, we employed bioinformatics and microarray techniques to identify 98 DEGs and 3 hub genes in AS and non-AS macrophages. These genes could serve as diagnostic and therapeutic biomarkers for AS.

## Abbreviations

AS: Atherosclerosis
ASCVD: Atherosclerotic cardiovascular disease
BP: Biological process
CC: Cell component
CD55: Complement decay-accelerating factor
CTRP1: C1q/TNF-related proteins-1
DEG: Differentially expressed gene
DYNC2H1: Dynein cytoplasmic 2 heavy chain 1
ER: Endoplasmic reticulum
GEO: Gene Expression Omnibus
GnRH: Gonadotropin-releasing hormone
GO: Gene Ontology
IL-1: interleukin-1
IL-6: interleukin-6
IRF2BP2: Interferon regulatory factor 2-binding protein 2
KDELR3: KDEL endoplasmic reticulum protein retention receptor 3
KEGG: Kyoto Encyclopedia of Genes and Genomes
LDL: Low-density lipoprotein
MCODE: Molecular Complex Detection
MF: Molecular function
PPI: Protein–protein interaction
STRING: Search Tool for the Retrieval of Interacting Genes
TNF-α: Tumor necrosis factor-α
TOR: Target of rapamycin
